# Retraction Note: Osteogenic differentiation of mesenchymal stem cells promotes c-Jun-dependent secretion of Interleukin 8 and mediates the migration and differentiation of CD4+T cells

**DOI:** 10.1186/s13287-025-04714-7

**Published:** 2025-10-09

**Authors:** Feng Ye, Jinteng Li, Peitao Xu, Zhongyu Xie, Guan Zheng, Wenjie Liu, Guiwen Ye, Wenhui Yu, Jiajie Lin, Zepeng Su, Yunshu Che, Zhaoqiang Zhang, Peng Wang, Yanfeng Wu, Huiyong Shen

**Affiliations:** 1 Department of Orthopedics, Sun Yat-sen Memorial Hospital, Sun Yat-sen University, 107# Yan Jiang Road West, Guangzhou, 510120 Guangdong China; 2 Department of Orthopedics, The Eighth Affiliated Hospital, Sun Yat-sen University, 3025# Shen Nan Middle Road, Futian District, Shenzhen, 518033 Guangdong China; 3 Center for Biotherapy, The Eighth Affiliated Hospital, Sun Yat-sen University, 3025# Shen Nan Middle Road, Futian District, Shenzhen, 518033 Guangdong China

**Retraction: Stem Cell Research & Therapy (2022) 13:58** 10.1186/s13287-022-02735-0

The Editors-in-Chief have retracted this article. After publication, the authors contacted the journal to request a correction to Fig. 6j, as the flow cytometry plot of the 10d group was mistakenly copied from the 10d group in Fig. 5c. However, further checks by the journal identified high similarity between Fig. 6f si-NC 14d and si-c-Jun 10d bottom images, as well as inconsistencies between the raw data files provided by the authors for validation and the published data.

The Editors-in-Chief therefore no longer have confidence in the presented data.

Jinteng Li, Zhongyu Xie, Yanfeng Wu and Huiyong Shen agree with this retraction. The other authors have not responded to any correspondence from the editor or publisher about this retraction.

